# Pyridoxamine Supplementation Effectively Reverses the Abnormal Phenotypes of Zebrafish Larvae With PNPO Deficiency

**DOI:** 10.3389/fphar.2019.01086

**Published:** 2019-09-20

**Authors:** Po-Yuan Chen, Hung-Chi Tu, Verne Schirch, Martin K. Safo, Tzu-Fun Fu

**Affiliations:** ^1^College of Medicine, Institute of Basic Medical Science, National Cheng Kung University, Tainan, Taiwan; ^2^Department of Medicinal Chemistry and Institute for Structural Biology, Drug Discovery and Development, School of Pharmacy, Virginia Commonwealth University, Richmond, VA, United States; ^3^Department of Medical Laboratory Science and Biotechnology, College of Medicine, National Cheng Kung University, Tainan, Taiwan

**Keywords:** pyridoxal 5′-phosphate (PLP)-dependent neonatal epileptic encephalopathy, pyridoxine 5′-phosphate oxidase, pyridoxamine, zebrafish, animal model

## Abstract

Neonatal epileptic encephalopathy (NEE), as a result of pyridoxine 5′-phosphate oxidase (PNPO) deficiency, is a rare neural disorder characterized by intractable seizures and usually leads to early infant death. The clinical phenotypes do not respond to antiepileptic drugs but are alleviated in most cases by giving large doses of pyridoxal 5′-phosphate (PLP). PLP is the active form of vitamin B6 participating in more than 100 enzymatic pathways. One of the causes of NEE is pathogenic mutations in the gene for human *PNPO* (h*PNPO*). PNPO is a key enzyme in converting pyridoxine (PN), the common dietary form of vitamin B6, and some other B6 vitamers to PLP. More than 25 different mutations in hPNPO, which result in reduced catalytic activity, have been described for PNPO-deficiency NEE. To date, no animal model is available to test new therapeutic strategies. In this report, we describe using zebrafish with reduced activity of Pnpo as an animal model. Knocking down zPnpo resulted in developmental anomalies including brain malformation and impaired locomotor activity, similar to the clinical features of PNPO-deficiency NEE. Other anomalies include a defective circulation system. These anomalies were significantly alleviated by co-injecting either z*pnpo* or h*PNPO* mRNAs. As expected from clinical observations in humans, supplementing with PLP improved the morphological and behavioral anomalies. PN only showed marginal positive effects, and only in a few anomalies. Remarkably, pyridoxamine (PM), another dietary form of vitamin B6, showed rescue effects even at a lower concentration than PLP, presenting a possible new therapeutic treatment for PNPO-deficiency NEE. Finally, GABA, a neurotransmitter whose biosynthesis depends on a PLP-dependent enzyme, showed some positive rescue effect. These results suggest zebrafish to be a promising PNPO-deficiency model for studying PLP homeostasis and drug therapy *in vivo*.

## Introduction

A severe neurological problem that presents in prenatal, neonatal, or infant children is neonatal epileptic encephalopathy (NEE) as a result of pyridoxine 5′-phosphate oxidase (PNPO) deficiency (Mills et al., 2005; Hoffmann et al., 2007; Ormazabal et al., 2008; Musayev et al., 2009; Balasubramaniam et al., 2010; Bowling, 2011; Ghatge et al., 2012; Mills et al., 2014; di Salvo et al., 2017a; di Salvo et al., 2017b; Wilson et al., 2019). Most patients with this condition are born prematurely and may die if not treated (Mills et al., 2005; Hoffmann et al., 2007). Apart from the typical severe and intractable seizures (tonic, myoclonic, and clonic), other expressed symptoms may include fetal distress, lactic acidosis, repetitive facial movement, hypoglycemia, stunted growth, anemia, increased blood lactate, electroencephalogram with burst suppression pattern, and asphyxia (Mills et al., 2005; Panayiotopoulos, 2005; Hoffmann et al., 2007; Ormazabal et al., 2008; Ghatge et al., 2012; Mills et al., 2014). The symptoms are in most part non-responsive to conventional antiepileptic drugs; however, surviving infants show improvement when treated with large and repeated doses (60–100 mg/kg/day) of PLP (Mills et al., 2005; Hoffmann et al., 2007; Ormazabal et al., 2008; Ghatge et al., 2012; Mills et al., 2014). In some instances, patients respond to pyridoxine (Mills et al., 2014; Plecko et al., 2014). Although PLP therapy has led to normal developmental outcomes in several patients, high doses of PLP are known to cause seizures (Ishioka et al., 1995; Hammen et al., 1998) and/or severe liver damage (Mills et al., 2012). This toxic effect of PLP is likely due to the reactive aldehyde forming complexes (aldimines) with non-PLP proteins and interfering with their function (Bartzatt and Beckmann, 1994; Ishioka et al., 1995; Salazar and Tapia, 2001; Scott et al., 2008). PLP is a cofactor for more than 100 vitamin B6 (PLP-dependent) enzymes that are involved in a large number of metabolic pathways, for example, amino acid metabolism, glucose metabolism, and heme and lipid syntheses (Percudani and Peracchi, 2009; di Salvo et al., 2011; Ghatge et al., 2012; di Salvo et al., 2017a). The function and development of the central nervous system also require enzymes that use PLP as a cofactor (Riggs et al., 1996; Musayev et al., 2009; Percudani and Peracchi, 2009; di Salvo et al., 2011; Garcia-Cazorla A and Clayton, 2012; Ghatge et al., 2012; Mills et al., 2012; Mills et al., 2014; di Salvo et al., 2017a). For example, the synthesis of many neurotransmitters, for example, GABA, serotonin, melatonin, dopamine, epinephrine, norepinephrine, and histamine, to mention a few, are dependent on several PLP-dependent enzymes (Riggs et al., 1996). Deficiency of PLP in cells is, therefore, likely to impact the development of many organs.

**Figure 1:**
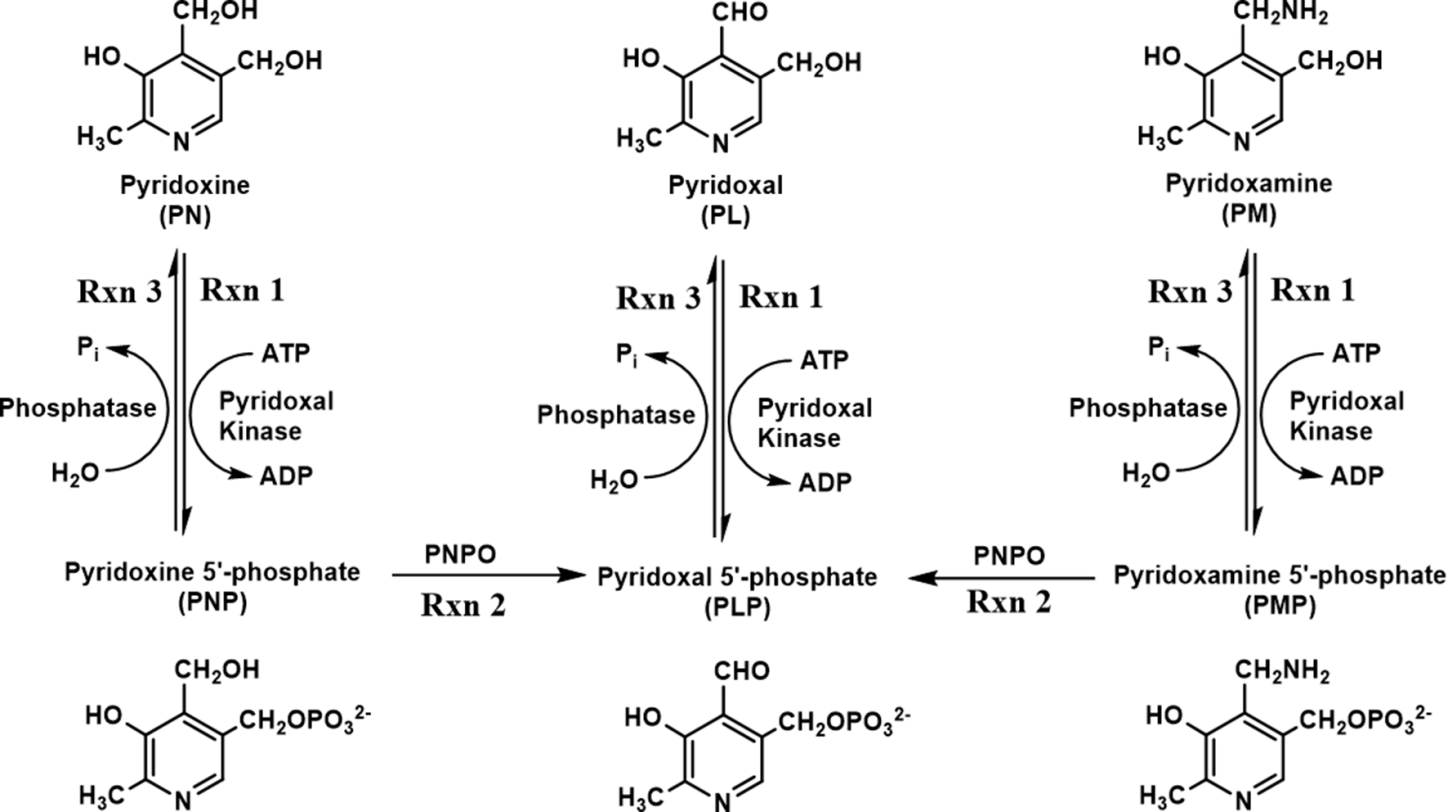
Schematic diagram of vitamin B6 metabolism. B6 vitamers, including pyridoxine (PN), pyridoxal (PL), and pyridoxamine (PM), can be phosphorylated to pyridoxine 5′-phosphate (PNP), pyridoxal 5′-phosphate (PLP), and pyridoxamine 5′-phosphate (PMP) by pyridoxal kinase. The phosphorylated form of B6 vitamers can also be hydrolyzed by pyridoxal phosphatase. The conversion of PNP/PMP to PLP requires an active pyridoxine 5′-phosphate oxidase (PNPO).

One form of NEE (PNPO-deficiency NEE) is caused by mutations in the human PNPO (h*PNPO*) gene encoding hPNPO. At present, about 25 different mutations have been reported (Mills et al., 2005; Hoffmann et al., 2007; Ormazabal et al., 2008; Musayev et al., 2009; Balasubramaniam et al., 2010; Bowling, 2011; Ghatge et al., 2012; Mills et al., 2014; di Salvo et al., 2017a; Wilson et al., 2019). Humans, unlike most prokaryotes, rely on a B6 salvage pathway that includes hPNPO, pyridoxal kinase, and PLP phosphatase to synthesize and/or recycle PLP during protein turnover (**Figure 1**) (di Salvo et al., 2011; di Salvo et al., 2012; Ghatge et al., 2012). The primary B6 forms, pyridoxine (PN), pyridoxamine (PM), and pyridoxal (PL), are phosphorylated to pyridoxine 5′-phosphate (PNP), pyridoxamine 5′-phosphate (PMP), and pyridoxal 5′-phosphate (PLP), respectively, by pyridoxal kinase (**Figure 1**). PNP and PMP are converted to PLP by PNPO. PLP is converted to PL by phosphatases (**Figure 1**) during protein turnover, which is then reconverted to PLP by pyridoxal kinase (**Figure 1**). Another type of B6-dependent epilepsy is due to biallelic variants in the enzyme antiquitin (ALDH7A1) and is referred to as PN-dependent epilepsy (Pena et al., 2017; Coughlin et al., 2019). Antiquitin catalyzes oxidation of α-aminoadipic semialdehyde to α-aminoadipic acid. Its inactivation by mutations leads to a metabolic buildup of piperideine-6-carboxylic acid that reacts with and depletes PLP from the cell. PN-dependent NEE is characterized by mild to severe clinical symptoms and usually responds to treatment with pyridoxine and/or conventional antiepileptic drugs (Pena et al., 2017; Coughlin et al., 2019; Wilson et al., 2019). Recently, mutations in the gene encoding a PLP-binding protein that is potentially crucial for B6 homeostasis (PLPHP or PROSC) have also been shown to cause a novel form of B6-dependent epilepsy (Johnstone et al., 2019).

Development of new therapies and animal models to study these various forms of vitamin B6 pathophysiology is warranted. This report focuses on using zebrafish as an animal model for studying PNPO-deficiency NEE. Zebrafish has prominently served as a vertebrate model for research in disease mechanisms and drug discovery. It shares about 70% similarity with human genetic and molecular pathways (Howe et al., 2013). Their low cost, easy maintenance, amenable genetic manipulation, high productivity, fast maturation, and transparent embryos have made these animals suitable for developmental studies, for example, for epilepsy or seizures (Baraban et al., 2005; Hortopan et al., 2010; Teng et al., 2011; Baraban et al., 2013; Grone et al., 2016; Sourbron et al., 2016; Griffin et al., 2017; Ibhazehiebo et al., 2018; O’Connor et al., 2019). Two major ways to induce seizure and/or other anomalies in zebrafish are genetic and pharmacologic (Baraban et al., 2005; Alfaro et al., 2011; Vermoesen et al., 2011; Pena et al., 2017; Vada et al., 2017; Yang et al., 2017). One genetic approach uses CRISPR/Cas9 gene editing to generate an aldh7a1-null zebrafish model that mimics the clinical and biochemical features of PN-dependent NEE (Pena et al., 2017). A recent report also showed that zebrafish larvae with PLPHP knockout displayed phenotypic signs of seizure and are responsive to treatment with pyridoxine (Johnstone et al., 2019).

In this study, we knocked down the expression of *z*Pnpo by injecting antisense morpholino oligonucleotides (MOs) to induce zPnpo deficiency in zebrafish. This resulted in several abnormal morphological features, including neuron damage, a defective circulation system at 3-day postfertilization (dpf), brain malformation at 2 dpf, spontaneous and erratic movements at 1 dpf, and premature death by 2 dpf. The rescue of the knockdown zPnpo morphants with both z*pnpo* and h*PNPO* mRNAs, and several forms of B6 vitamers were also studied.

## Materials and Methods

### Fish (*Danio rerio*) Care and Maintenance

Zebrafish (*Danio rerio*, AB strain) were purchased from NTHU-NHRI Zebrafish Core Facility, Taiwan. They were bred and maintained at 28.5°C in a 10- to 14-h light–dark diurnal cycle following standard procedure (Westerfield, 2000). Both transgenic lines Tg(*sox10*:eGFP) and Tg(*cmlc2*:eGFP) were purchased from Taiwan Zebrafish Core Facility at ZeTH (support by MOST 105-2319-B-400-001). All usage and experiments, including adult and larval zebrafish, were approved by the Institutional Animal Care and Use Committee, National Cheng Kung University, Tainan, Taiwan (IACUC Approval No. 106086).

### Knockdown and Rescue of zPnpo Expression With MO and mRNA

Knockdown of zPnpo expression was accomplished by injecting embryos with the MO, specifically targeting the translation start site of z*pnpo* to block protein translation. The sequence of zebrafish Pnpo MO designed by the manufacturer (Gene-Tools, LLC, Philomath, OR) based on the zebrafish *pnpo* coding sequence (NM_001256178.1) is 5′-ACGTCTCATGCTTGTTCCGCG-3. A scrambled MO mixture containing 4^25^ different nucleotide sequences was used as a standard control MO. For microinjection, approximately 4.6 nL of solution containing 5 to 10 ng of zPnpo MO or 10 ng of scrambled MO was injected into embryos at the one- to two-cell stage. For rescuing with mRNA, MOs were co-injected with 800 pg of z*pnpo* or h*PNPO* mRNA. All reagents for microinjection were dissolved in degassed and RNase-free Danieu’s buffer to make proper stock solutions.

### zPnpo Protein Expression

The expression of zPnpo was examined with Western blotting as previously described, with minor modifications (Kao et al., 2008). In brief, 30 embryos at 24 hpf were homogenized in 30 μL of E buffer (40 mM of Tris–HCl, 2 mM of EDTA, 2 mM of EGTA, 500 mM of NaCl, and 4 M of urea) containing 10 mM of phenylmethanesulfonyl fluoride (Sigma, 78840) and proteinase inhibitor cocktail (Sigma, catalog No. P8340). After centrifugation, 30 µg of total protein in the supernatant was subjected to Western blot analysis with rabbit anti-zPnpo antibodies (Genetex, customized).

### Whole-Mount *In Situ* Hybridization (WISH)

WISH was performed following the standard protocol as previously described, with minor modifications (Tu et al., 2017). In brief, riboprobes against zebrafish *pnpo* transcripts (anti-sense) and template strand (sense) *pax2.1* transcripts were synthesized *in vitro* from the plasmid containing z*pnpo* coding sequence linearized with *Nco*I (using SP6 polymerase) and *Nde*I (using T7 polymerase) and the plasmid containing *pax2.1* coding sequence linearized with *Nde*I (using T7 polymerase), respectively, in the presence of digoxigenin (DIG) using the DIG-labeled RNA kit (Roche, catalog no. 11175025910).

### Cryosection and Hematoxylin and Eosin (H&E) Staining

The preparation and H&E staining for zebrafish tissue cryosections were performed following the protocols in *The Zebrafish Book* (Westerfield, 2000).

### RNA Isolation and Reverse Transcription–Polymerase Chain Reaction (RT-PCR)

Total RNA was extracted from 30–40 embryos at indicated stages as previously described (Kao et al., 2014). The z*pnpo* transcript was analyzed with RT-PCR with forward primer 5′-CAGCATCAAGCAGAGGGAG-3′ and reverse primer 5′-AACGCAGGACATTGAGGA-3′. Actin was used as an internal control with forward primer 5′-AGACATCAAGGAGAAGCTGTG-3′ and reverse primer 5′-TCCAGACGGAGTATTTAC-3′.

### Compound Treatment

B6 vitamers, including PLP, PN, PM, and PL, were freshly prepared in embryo water and were added to embryo water at 1 hpf to reach the highest concentrations without inducing apparent harmful effects to embryonic development for rescue. The concentrations used were 1 mM for PN and 0.1 mM for PM and PL for all trials. The neurotransmiter GABA was added to embryos water at 6 hpf to reach the concentration of 0.5 mM. Considering the toxicity, stability, and light sensitivity, the PLP concentrations used were sequentially decreased until the indicated stages: 1 mM of PLP at 0–1 dpf, 0.5 mM at 1–2 dpf, and 0.1 mM at 2–4 dpf. Embryos were continuously exposed to the tested B6 vitamers with larval morphology and survival recorded at the indicated times. Embryo water was replaced daily with water containing freshly prepared B6 vitamers.

### Cloning, Expression, and Purification of Recombinant zPnpo

The recombinant zPnpo was obtained following the protocol as previously described (Kao et al., 2008). The forward primer (5′-GGATCCAGACGTCTGTTAAGGTTTTG-3′) and reverse primer (5′-CAAGCTTTCAAGGGGACAAGCG-3′), with design based on the *zpnpo* coding sequence (NM_001256178), were used for PCR cloning of the z*pnpo* coding sequence from the zebrafish cDNA library. The 847-bp PCR product was cloned into the expression vector pET43.1a *via Bam*HI and *Hin*dIII restriction enzyme sites. The recombinant zPnpo was overexpressed with a Nus-His tag at the N-terminus in *Escherichia coli* strain HMS174(DE3) and purified with a nickel Sepharose column. The purified recombinant zPnpo fusion protein was digested with thrombin to remove the Nus-His tag before being subjected to antibody production. The specificity of the antibodies was confirmed by comparing the Western blotting signals generated from the *E. coli* extracts with/without containing the induced recombinant zPnpo that was used for antibody generation (**Suppl. S1**).

### Spontaneous Movement

Larvae at 1 dpf were placed on a concave slide with embryo water under a transmitted-light stereomicroscope (Leica, MDG28) equipped with a digital single-lens reflex camera (Canon, EOS 550D). Larval spontaneous contraction was video-recorded in 60 frames per second and analyzed with EthoVision XT (Noldus, Wageningen, The Netherlands).

### Larval Swimming Behavior Analysis

Larval swimming behavior was recorded and analyzed with a high-throughput image recording and analysis system from DanioVision^™^ (Noldus, Wageningen, The Netherlands). The larva at 4 dpf was placed in a well on a 48-well plate. The plate was moved into the DanioVision^™^ device 15 min before analysis to allow larvae to rest. Larval swimming behavior was video-tracked and recorded for 1 h at 9.94 frames per second. The velocity, maximum acceleration, relative turning angle, absolute turning angle, and body activity track of movement were analyzed with the built-in analytic software EthoVision XT.

### Statistical Analysis

The statistical significance was calculated with one-way analysis of variance (ANOVA) (Kruskal–Wallis test) for behavior study and one-tailed Mann–Whitney nonparametric *U* test for phenotype analysis at 95% confidence intervals using the software GraphPad Prism 5.

## Results

### Zebrafish Pnpo Displays Structural Conservation During Evolution

**Figure 2:**
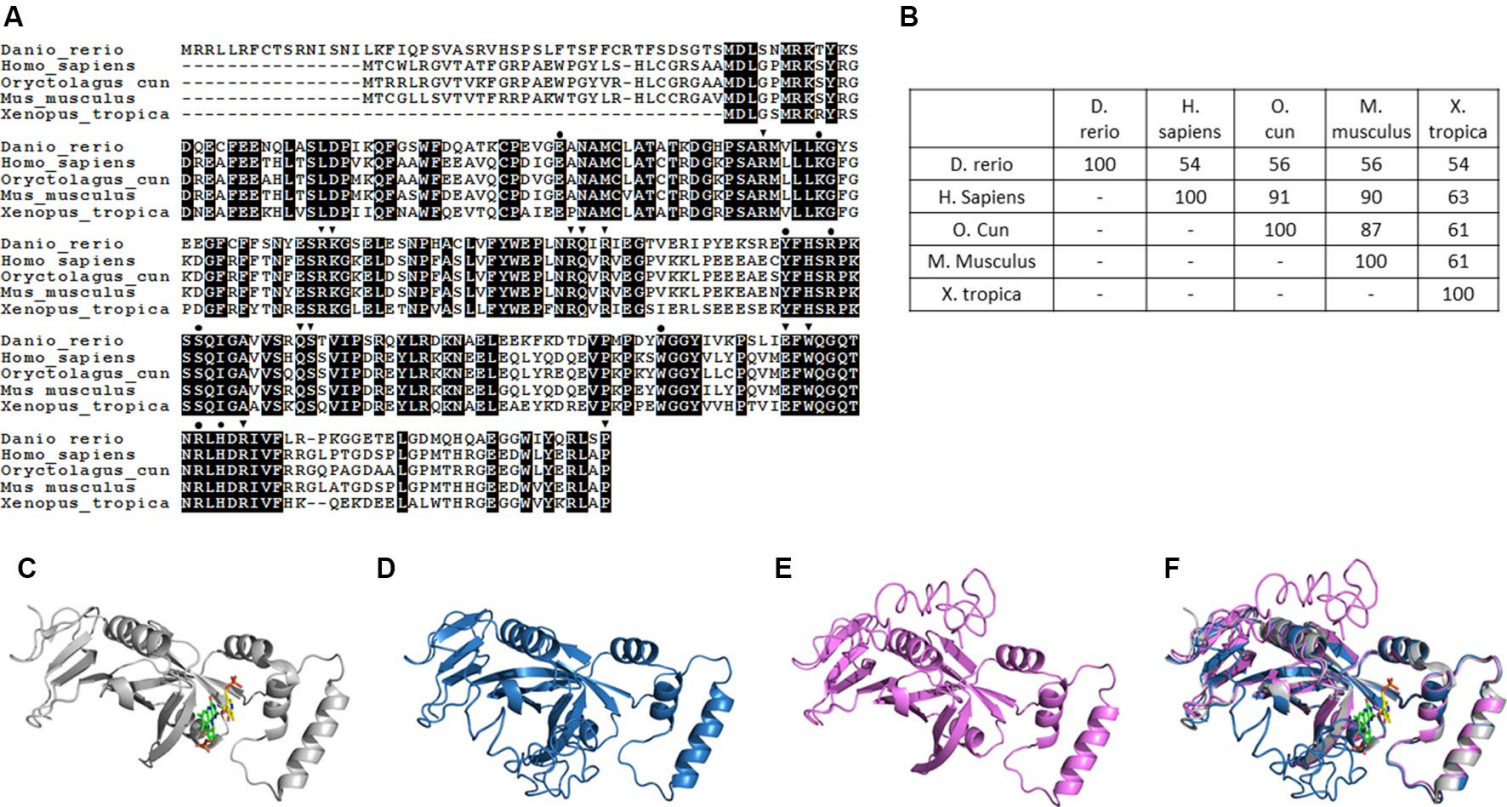
Structural and phylogenic comparison of zebrafish Pnpo with enzymes from four different sources. The amino acid sequences of PNPOs from the indicated species were analyzed and compared for functional domains and evolutionary conservation. **(A)** The peptide sequences of PNPOs from the indicated species were aligned. The shaded letters indicate the amino acids that are identical among compared species. Dots indicate the PNP/PLP binding sites, and arrowheads indicate the FMN binding sites. **(B)** A phylogenetic table shows the identity between compared enzymes, revealing the evolutionary relationships of PNPOs among the compared species. **(C)** The crystal structure of experimental hPNPO (Musayev et al., 2003), **(D)** the simulated structures of hPNPO, and **(E)** zPnpo are shown in ribbon diagram. **(F)** Superposed experimental hPNPO crystal structure (grey) and simulated zPnpo (magenta) and hPNPO (cyan) structures. The simulated structures are obtained using I-TASSER (http://zhanglab.ccmb.med.umich.edu/I-TASSER/). The compared peptide sequences include zebrafish Pnpo (NP_001243107.1); human PNPO (NP_060599.1); rabbit PNPO (XP_002719371.1); mouse PNPO (NP_598782.1); and *Xenopus* Pnpo (NP_001120016.1). PNPO, pyridoxine 5′-phosphate oxidase; PLP, pyridoxal 5′-phosphate.

Pnpo has been identified in many species, with zebrafish displaying conservation in both amino acid sequence and folding structure during evolution. To our knowledge, there is only one copy of z*pnpo* in the zebrafish genome, even though many genes in zebrafish exist with extra copies. The z*pnpo* gene contains seven exons located on chromosome 12 and encodes zPnpo, which has 267 amino acids (**Figure 2A**). The peptide sequence alignment and comparison show that zPnpo is 54% identical and 71% homologous to hPNPO (**Figures 2A, B**). In addition, the key residues in hPNPO for binding PNP/PLP (E77, W206, H227, K100, Y157, R161, R225, and S165) and FMN (Q174, Q139 W219, P261, S175, R116, R141, R95, W219, E217, K117, R138, and R229) are strictly conserved in zPnpo (**Figure 2A**). Our group had previously determined the crystal structure of hPNPO (**Figure 2C**) (Musayev et al., 2003). As expected from the high sequence similarity between hPNPO and zPnpo (**Figures 2A, D, E**), the two structures predicted by the online program I-TASSER (Iterative Threading ASSEmbly Refinement) showed almost identical predicted folds that are also close to the determined fold for hPNPO (**Figure 2F**) (Zhang, 2008; Roy et al., 2010; Yang et al., 2015). These data on the structural analysis of PNPO support the conservation among PNPO during evolution.

### The Expression of *pnpo* Is Ubiquitous and Essential in Zebrafish and for Embryogenesis

**Figure 3:**
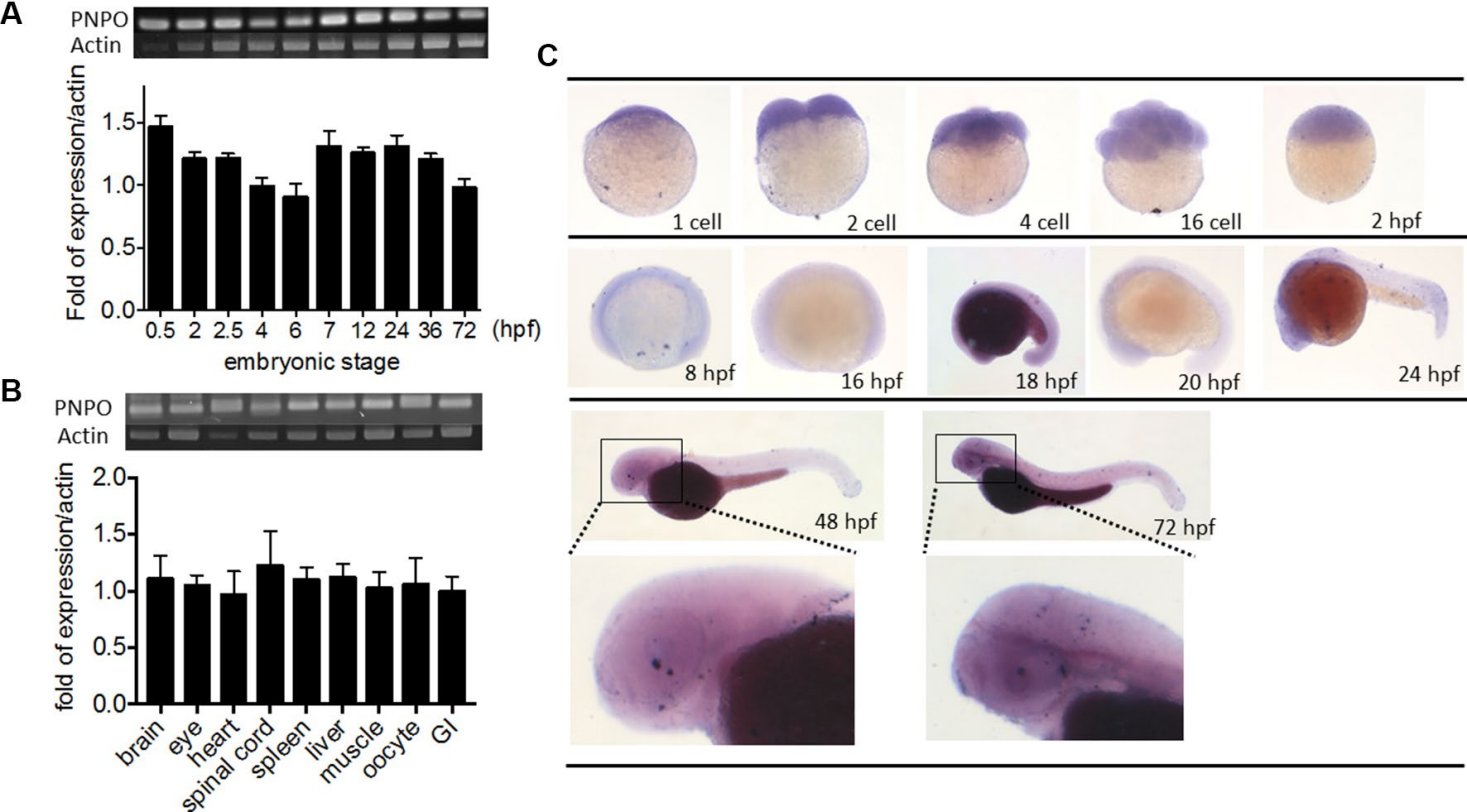
Spatial and temporal distribution of zebrafish *pnpo*. The expression profiles of *pnpo* in zebrafish at different developmental stages and tissues in adult fish were examined by RT-PCR and WISH. Embryos at the **(A)** indicated stages and **(B)** tissues of adult female zebrafish were extracted for total mRNA and subjected to RT-PCR with z*pnpo*-specific primers. The amount of z*pnpo* transcripts was normalized with zebrafish *β*-*actin*. Results presented are the averages shown in mean ± SEM from at least three independent repeats. **(C)** Embryos at various developmental stages were examined with WISH by probing with the DIG-labeled riboprobes specific to z*pnpo* as described in the Materials and Methods. RT-PCR, reverse transcription–polymerase chain reaction; WISH, whole-mount *in situ* hybridization; DIG, digoxigenin.

We examined the temporal expression and spatial distribution of z*pnpo* in zebrafish. RT-PCR of cDNA collected from the embryos at early developmental stages revealed that z*pnpo* was first detected at the one- to two-cell stage and remained detectable at least to 3 dpf (**Figure 3A**). Results of RT-PCR using the cDNA templates collected from different tissues of adult female zebrafish also showed ubiquitous z*pnpo* expression (**Figure 3B**). No significant difference was found in the tissue distribution of *zpnpo* transcripts between female and male fish (data not shown). Images from WISH with the anti-sense riboprobe specific to z*pnpo* showed that z*pnpo* transcripts were ubiquitously distributed in embryos from one- to two-cell stage to 3 dpf, and with higher expression in the brain region at 20–24 hpf (**Figure 3C**). No appreciable signal was observed in the embryo samples with the sense riboprobe, further confirming the spatial and temporal expression of z*pnpo* during embryogenesis (**Suppl. S2**). These results suggest the essentialness of Pnpo for zebrafish embryogenesis.

**Figure 4:**
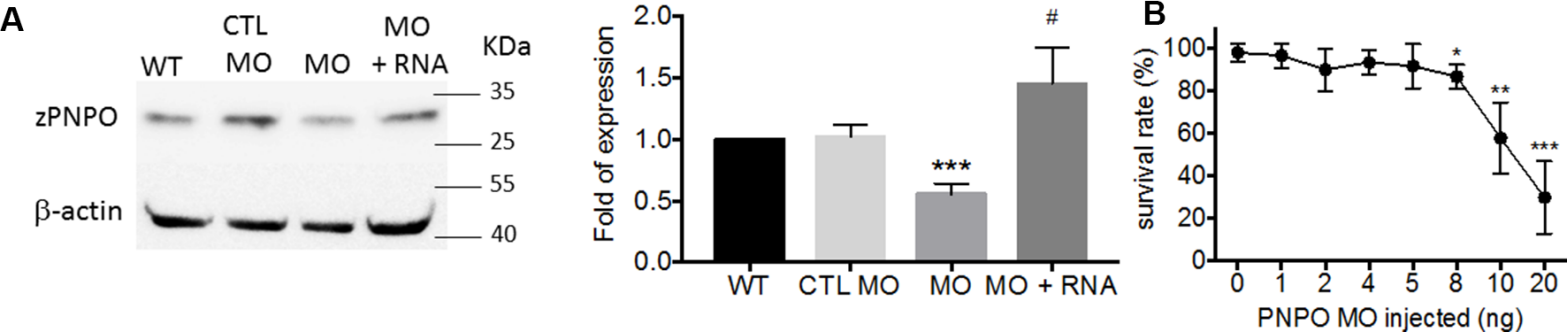
The impact of knocking down zPnpo. **(A)** The protein levels of zPnpo in zebrafish embryos injected with 10 ng of scrambled control MO (CTL MO; lane 2), 10 ng of zPnpo MO (MO; lane 3), or zPnpo MO plus 800 pg of zebrafish *pnpo* mRNA (MO + RNA; lane 4) at 14 hpf and wild-type embryos of the same stage (WT; lane 1) were analyzed with Western blotting (left) and quantified by normalizing with β-actin (right). Shown here are the representative image and average of at least five independent repeats. **(B)** Dose-dependent decreased survival was observed in zPnpo morphants at 3 dpf. Data presented were the representatives and averages of at least three independent repeats with *n* = 40∼60 embryos for each group and expressed as mean ± SEM. The statistical significance was calculated with one-tailed Mann–Whitney nonparametric *U* test by comparing the experimental groups with wild type (*) or with zPnpo MO (#). *^/#^p < 0.05; **p < 0.01; ***p < 0.001. MO, morpholino oligonucleotide.

**Figure 5:**
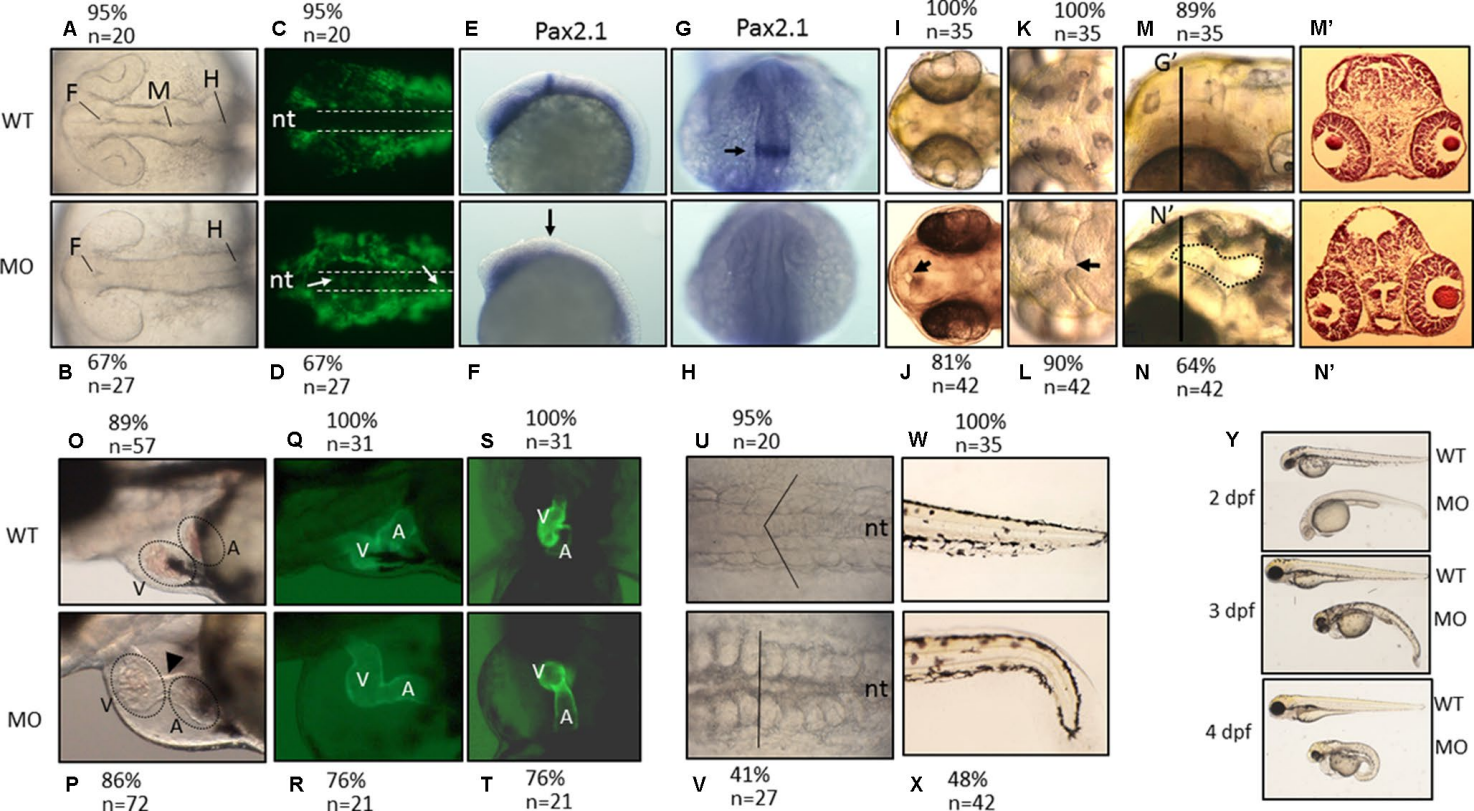
The morphological characteristics of zPnpo morphants. Zebrafish embryos of wild-type and transgenic lines injected with *z*Pnpo MO at one- to two-cell stages were grown in embryo water and recorded for embryonic organogenesis and development. Tg(*sox10*:eGFP) embryos with/without MO injection were imaged at 18 hpf under dissecting light and fluorescent microscopes **(A**–**D)** and also subjected to WISH analysis with riboprobes *pax2.1* **(E**–**H)**. Migratory neural crest cells (green fluorescent signals pointed by white arrows) were absent in the closing neural tube (dashed line) of wild-type larvae but remained in that of morphants. Lack or significantly reduced signal for *pax2.1* (arrows) were also observed in zPnpo morphants. Both wild-type and zPnpo morphants at 2 dpf were imaged for brain region **(I**–**N)**. A crater-like opening was observed in diencephalic ventricle (arrows) and hindbrain (circled area) of zPnpo morphants. H&E-stained cryosections prepared from the head region [indicated by vertical lines in **(N)**] of 2-dpf morphants revealed a cavity (*), which was not observed in wild-type larvae **(M’** and **N’)**. Tg(*cmlc2*:eGFP) embryos with/without MO injection were imaged **(O**–**R)** and video-recorded (**S** and **T**, the representative still frames from **Suppl. 3–10**) under a fluorescent microscope for heart development at 3 dpf. A tubing heart with incorrectly positioned ventricle and atrium were apparent in zPnpo morphants. The somite formation **(U** and **V)** and the development of trunk and tail **(W** and **X)** were recorded at 18 hpf and 3 dpf, respectively. The somites with chevron shape (solid line in **U**) can be seen in wild-type embryo. The full view of larvae at the indicated stages was imaged from the lateral view to show the overall morphology and size, which also revealed apparent body curvature for zPnpo morphants **(Y)**. For the images to be taken, embryos/larvae of the indicated stages were removed from the embryo water and placed individually on a drop of methylcellulose for photographing. All images were taken with anterior to the left for lateral view except for brain **(A**–**D**, **E**–**L)** and somites **(U**–**V)** (dorsal) and video recording **(S** and **T)** (ventral). WT, wild-type; MO, zPnpo morphants; F, forebrain ventricle; M, midbrain ventricle; H, hindbrain ventricle; nt, neural tube; V, ventricle; A, atrium; WISH, whole-mount *in situ* hybridization; H&E, hematoxylin and eosin.

We found that PNPO is essential for organogenesis and embryonic development. To investigate the biological significance of zPnpo during embryogenesis, the MO specific to the translation start site of z*pnpo* mRNA was injected into the zebrafish embryos at the one- to two-cell stage to decrease the expression of zPnpo, resulting in zPnpo morphants. The quantitative results of Western blotting analysis revealed significantly decreased zPnpo protein levels in morphants, confirming the knockdown effects (**Figure 4A**). The survival rates of morphants, recorded at 3 dpf, were dose dependently lowered (**Figure 4B**). Abnormal morphology and defect of several organs/tissues, including small eyes, malformed swim bladder, body curvature, tubing heart, and pericardial edema, were also observed in zPnpo morphants (**Figure 5**). In addition, hindered development of brain ventricles was observed (**Figures 5A, B**). The wide spectrum of the tissues affected by zPnpo knockdown implied that neural crest cells (NCCs), the rudiment of a myriad of organs/tissues, might be affected. Therefore, we also knocked down zPnpo expression in the embryos of Tg(*sox10*:eGFP), the transgenic fish expressing green fluorescent protein specifically in all NCCs. Unlike in control larvae where most NCCs had evacuated from the dorsal margin of closing neural tube to distant sites with reduced distribution at the bilateral bands, migratory NCCs in zPnpo morphants were still present and remained in and around the neural tube (**Figures 5C, D**). This impeded NCC migration/distribution was reflected in the subsequent obstruction on the development of NCC-derived tissues, especially the impaired brain and midbrain–hindbrain boundary (MHB). Further characterization on the development of brain and nervous system was conducted by WISH with the riboprobes for the neural marker *pax2.1* (the markers for MHB). The results showed that the signal corresponding to *pax2.1* was lost or significantly reduced when zPnpo was knocked down (**Figures 5E–H**). In addition, distinct structures observed in the brain region of zPnpo morphants under light microscope revealed a crater-like opening in the diencephalic ventricle and midbrain–hindbrain of 2-dpf morphants, indicating an abnormal closure of the ventricle and neural tube (**Figures 5I–N**). H&E-stained cryosections prepared from the brain region of zPnpo morphants revealed the presence of a cavity, which was not observed in wild-type larvae of the same stages of development (**Figures 5M’, N’**). These results further support impeded development of brain and nervous system in zPnpo morphants.

PNPO is crucial to heart and trunk development. zPnpo morphants displayed a tubing heart with enlarged cardiac chambers accompanied by pericardial edema, suggesting an unsuccessful looping of the heart tube (**Figures 5O, P**). The mispositioned ventricle and atrium were more evidently observed in the zPnpo morphants generated from the embryos of the transgenic line Tg(*cmlc2*:eGFP), which express green fluorescent protein specifically in the heart (**Figures 5Q–T**). Lowered heart rate and obstructed blood flow with diminished circulating blood cells were also apparent, indicating a defective circulation system (**Suppl. S3–S10**). Normally, embryonic somites will start to elongate into muscle segments at 16–18 hpf and take on a chevron shape with the notochord intersecting the apexes of the v-shaped segments. Conversely, these v-shaped segments in zPnpo morphants were disrupted and became cuboidal (**Figures 5U, V**). In addition, the notochord was misshaped with indistinct borders. zPnpo morphants also displayed body curvature and malformed tails and fins (**Figures 5W, X**). The presence of “body curvature” and “no tail” often signals “mild” and “severe” defects of larval trunk development, respectively (**Suppl. S11**). Developmental delay was also observed in zPnpo morphants. Occasionally, some morphants ceased their development at around 12 hpf. Most of the morphants continue to develop but appear to be smaller in size than the control and wild-type larvae of the same stage (**Figure 5Y**). Decreased head–trunk angle was also found in zPnpo morphants, indicating developmental delay.

**Figure 6:**
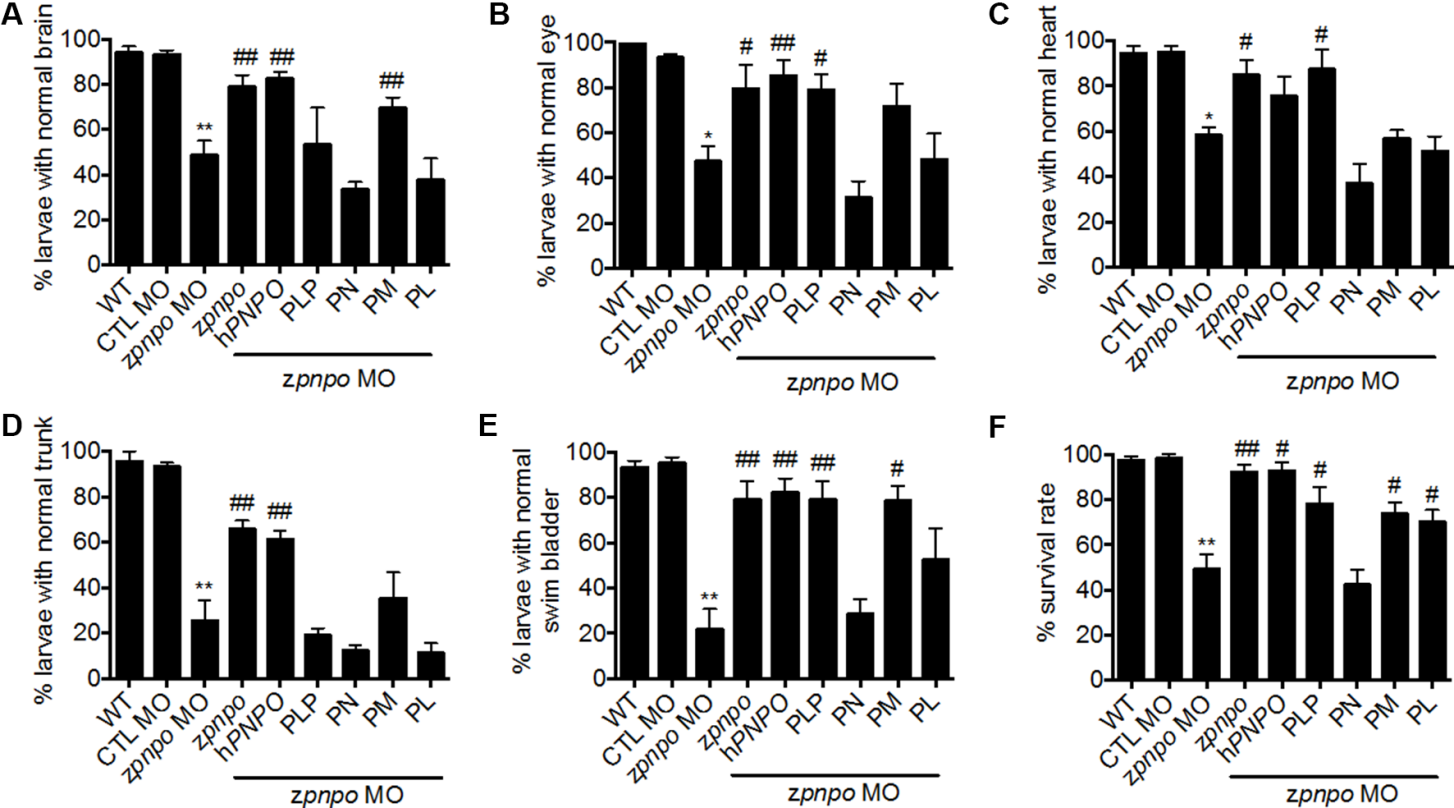
Rescue of zPnpo morphant morphology. Embryos injected with 5 ng of zPnpo MO at one- to two-cell stage were co-injected with 800 pg of z*pnpo* or h*PNPO* mRNA (columns 4 and 5) or grown in embryo water containing 1 mM of PLP, 1 mM of PN, 0.1 mM of PM, or 0.1 mM of PL (columns 6–9). Embryos injected with 10 ng of scrambled control MO were used as control (CTL). Development of various tissues was examined under light dissecting microscope at 3 dpf for brain **(A)**, eyes **(B)**, and heart **(C)**; at 4 dpf for trunk (body curvature) **(D)**; and at 5 dpf for swim bladder **(E)**. The larvae, displaying the morphology comparable with that of wild-type control of the same stage for the characterized tissue/organ, were categorized as normal, and the percent of larvae displaying normal morphology in each group was recorded. **(F)** The survival rate of zPnpo morphants was recorded at 4 dpf. The averages of data are reported from at least three independent repeats with *n* = 60∼80 embryos for each group and expressed as mean ± SEM. The statistical significance was calculated with one-tailed Mann–Whitney nonparametric *U* test by comparing the experimental groups with wild type (*) or with zPnpo MO (#). “*^/#^p < 0.05; **^/##^p < 0.01. MO, morpholino oligonucleotide; PLP, pyridoxal 5′-phosphate; PN, pyridoxine; PM, pyridoxamine; PL, pyridoxal.

**Figure 7:**
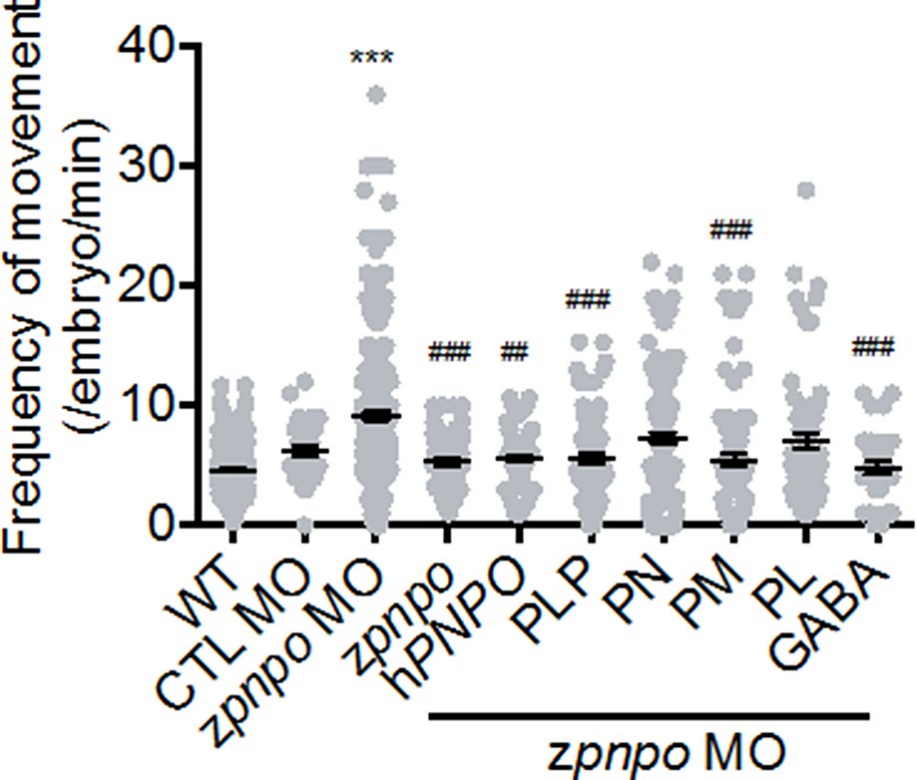
The locomotor activity of zPnpo morphants at 24 hpf. Embryos injected with 5 ng of zPnpo MO at one- to two-cell stages were co-injected with 800 pg of z*pnpo*/h*PNPO* mRNA (columns 4 and 5) or grown in embryo water containing 1 mM of PLP, 1 mM of PN, 0.1 mM of PM, 0.1 mM of PL, or 0.5 mM of GABA (columns 6–10) and recorded for larval spontaneous contraction at 24 hpf. Embryos injected with 10 ng of scrambled control MO were used as control (CTL). Reported are the averages of data from at least three independent repeats with *n* = 80∼120 embryos for each group and expressed as mean ± SEM. The statistical significance was calculated with one-way ANOVA (Kruskal–Wallis test) by comparing the experimental groups with wild type (*) or with zPnpo MO (#). ^##^ *p* < 0.01; ***/^###^ *p* < 0.001. MO, morpholino oligonucleotide; PLP, pyridoxal 5′-phosphate; PN, pyridoxine; PM, pyridoxamine; PL, pyridoxal; ANOVA, analysis of variance.

To determine if these developmental abnormalities were the result of off-target effects or non-specific toxicity, we co-injected either human or zebrafish *pnpo* mRNAs with MO to embryos at the one- to two-cell stage. Our results showed that both human and zebrafish mRNAs attenuated the mortality and severity of anomalies observed in zPnpo morphants, substantiating knockdown specificity (**Figures 6**, **7** and **Suppl. S11**). These data further support that zPnpo activity is crucial to organogenesis during embryonic development. Our results also demonstrated that hPNPO can function as a replacement for zPnpo during embryonic morphogenesis.

### The Mortality and Developmental Anomalies Observed in zPnpo Morphants Is B6 Responsive

Different vitamers of B6 possess diverse efficacy in increasing larval survival and relieving the anomalies occurring to zPnpo morphants. To confirm the B6-deficient status due to PNPO knockdown and evaluate the rescuing effects of different B6 vitamers, PLP, PN, PM, and PL at the highest concentrations without inducing apparent harmful effect to embryonic development were used for rescue. PLP rescued the malformation of the eye, heart, and swim bladder. PM also rescued the malformation of the brain, eye, and swim bladder, while PL rescued the malformation of the swim bladder (**Figures 6A–E**). PN could not rescue any anomaly observed in the morphants. It was quite interesting that PM showed rescue effects, since PN and PM are unlikely to be metabolized into PLP due to the knockdown of zPnpo expression. This noteworthy observation, which will be discussed later, is likely due to reduced need for PLP in the cell because of the increased production of PMP by pyridoxal kinase from PM. As for larval survival, all reagents examined significantly increased morphant survival except PN (**Figure 6F**).

### Knocking Down zPnpo Affected Larval Locomotor Activity

In addition to effects on morphogenesis, the impact of zPnpo knockdown on larval spontaneous movement was evaluated. Zebrafish larvae exhibit spontaneous contractions at as early as 18 hpf. This spontaneous movement in early embryos represents embryonic locomotor activity, reflecting the integrity of the central nervous system and skeleton/muscle development. Our results showed that embryonic spontaneous movement was significantly increased in zPnpo morphants at 1 dpf (**Figure 7**, column 2). This increased movement indicates a seizure-like behavior. Morphant embryos co-injected with either z*pnpo* or h*PNPO* mRNA exhibited movement only slightly higher to that of wild-type embryos (**Figure 7**, columns 3 and 4), suggesting that the seizure-like movement was mostly corrected by expressing either zPnpo or hPNPO. Movement of morphants was monitored when embryos were grown in several forms of B6 vitamers (columns 5–8). The larvae grown in the presence of PLP or PM were similar to wild type in movement (columns 5 and 7), as well as the morphants co-injected with either z*pnpo* or h*PNPO* mRNAs. Morphants grown in the presence of either PN or PL showed an intermediate movement between wild type and morphants (columns 6 and 8). The slight improvement with PN probably reflects some residual zPnpo activity in the knockdown embryos. The presence of GABA in embryos’ water also significantly reversed the increased spontaneous movement observed in *z*Pnpo morphants (column 9).

**Figure 8:**
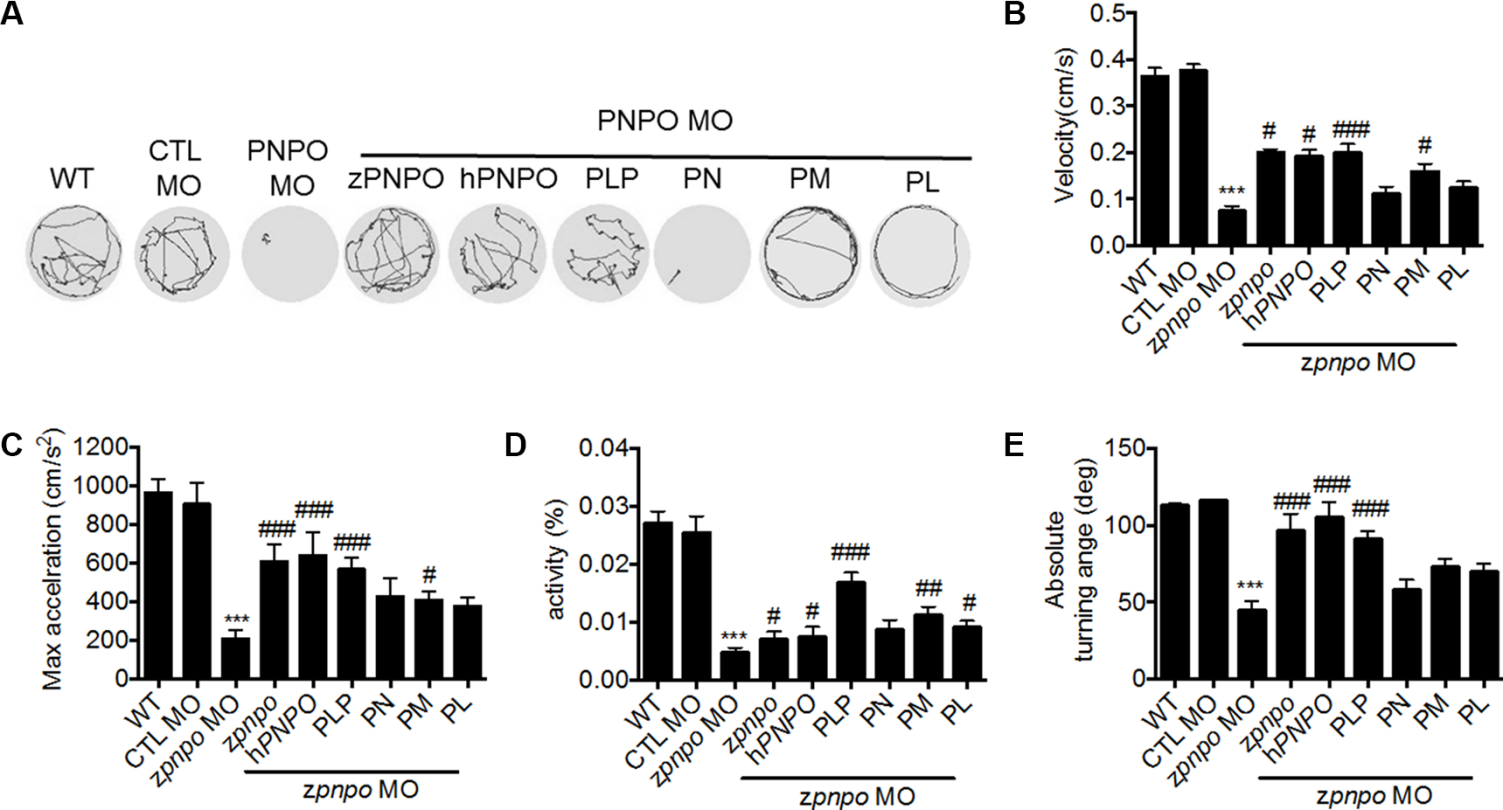
The locomotor activity of zPnpo morphants at 4 dpf. Zebrafish larvae with the indicated treatment were placed individually in each well on a 48-well plate and rested in the tested chamber for 5 min before recording. **(A)** The swimming track, representing larval cumulative location in 1 min, was recorded. **(B**–**E)** Larval swimming behavior was recorded for 30 min and analyzed for velocity **(B)**, maximum acceleration **(C)**, body activity **(D)**, and absolute turning angle **(E)** with the built-in analytic software in DanioVision High-throughput tracking system. Reported are the averages of data from at least three independent repeats with *n* = 30∼164 embryos for each group and expressed as mean ± SEM. The statistical significance was calculated with one-way ANOVA (Kruskal–Wallis test) by comparing the experimental groups with wild type (*) or with zPnpo MO (#). ^#^ *p* < 0.05; ^##^ *p* < 0.01; ***/^###^ *p* < 0.001. MO, morpholino oligonucleotide; ANOVA, analysis of variance.

Examination of the swimming behavior of zPnpo morphants after 4 dpf was conducted to evaluate larval locomotor activity. In **Figure 8A** are shown the recorded swimming tracks of wild type and morphants under different rescue conditions as described in **Figure 6**. The results are determined for velocity, acceleration, activity, and turning angle (**Figures 8B–E**). The deleterious effects of decreasing zPnpo expression caused by knockdown became more apparent during the later stages of the morphants growth. The morphant embryos have gone from increased movement at 1 dpf to almost no movement at 4 dpf. Morphants injected with either z*pnpo* or h*PNPO* mRNAs show improvement in movement parameters, but this treatment does not fully rescue the morphants (**Figures 8A–E**). These results confirm zPnpo knockdown specificity and suggest that organ damage over time has not been completely restored by any of the conditions tested. In these 4-day exposure studies, growth in the presence of B6 vitamers shows that embryos in PLP and PM provide the most effective protection for zPnpo knockdown embryos.

## Discussion

Mutations in hPNPO result in severe seizures in newborn children, which is described as PNPO-deficiency NEE (Mills et al., 2014; Plecko et al., 2014). Most patients only respond to the cofactor PLP as a treatment (Mills et al., 2005; Hoffmann et al., 2007; Ormazabal et al., 2008; Ghatge et al., 2012; Mills et al., 2014), while only a minor subset of patients also respond to PN therapy (Mills et al., 2014; Plecko et al., 2014). The observation that PLP is the normal therapy for PNPO-deficiency, while PN does not work in the majority of cases, is understood in the context of the salvage pathway for B6 vitamers found in most, if not all, eukaryotic organisms (see **Figure 1**) (di Salvo et al., 2011; di Salvo et al., 2012; Ghatge et al., 2012). Pyridoxal kinase converts PN, PM, and PL to their respective phosphorylated forms. PL is converted into PLP, which is required to convert newly synthesized apo-B6 enzymes to their catalytic active holo-enzyme form. Both PNP and PMP require the activity of PNPO to form PLP, explaining why PLP is the standard therapy for PNPO-deficiency NEE. However, the large doses of PLP used in therapy (60–100 mg/kg/day) (Mills et al., 2005; Hoffmann et al., 2007; Ormazabal et al., 2008; Ghatge et al., 2012; Mills et al., 2014) are known to have toxic side effects due to the reactive aldehyde of PLP forming aldimines with amino groups of proteins (N-terminal and lysyl residues), resulting in malfunction of these proteins (Bartzatt and Beckmann, 1994; Ishioka et al., 1995; Salazar and Tapia, 2001; Scott et al., 2008).

Our previous extensive work on the enzymes in the salvage pathway suggested to us that there are possible additional methods for treatment in addition to PLP (di Salvo et al., 2011; di Salvo et al., 2012; Ghatge et al., 2012). To test these ideas, we needed an animal model, and studies on other genetic diseases suggested that zebrafish would be less expensive and faster for this purpose. Zebrafish contains a single gene for zPnpo, with both amino acid sequence and structure being very similar to hPNPO, which is evidence that it is a good animal model for PNPO-deficiency NEE. In the current study, we show that the zebrafish z*pnpo* mRNA is distributed globally in developing embryos and ubiquitously among organs in adult fish (**Figure 3**). This is in agreement with the findings that the expression of h*PNPO* mRNA and hPNPO is ubiquitous in humans with liver and kidney displaying the highest levels of expression (Kang et al., 2004). The global expression of z*pnpo* reflects the importance of this gene in embryogenesis, which is further supported by the developmental delay and the anomalies occurring in a wide spectrum of organs/tissues observed in zPnpo morphants (**Figures 5**–**8**). In addition, the impeded morphological phenotypes and the resulting abnormal behavior observed in zPnpo morphants have recapitulated some of the clinical features observed with PNPO-deficiency NEE patients. Knocking down PNPO is expected to decrease PLP concentration in cells and affect amino acid, heme, DNA/RNA, neurotransmitter, and sugar metabolism, which will potentially interfere with cell proliferation and differentiation. There is a possibility that the observed anomalies were the consequence of the impeded embryonic primordium and germ layer formation at early stages.

The severity of abnormalities due to decreased zPnpo expression appears to be organ dependent, which may reflect the differences of zPnpo importance in each organ. The tail and swim bladder were affected the most (**Figures 6D, E**). The morphants also showed a defective circulation system, which has not been reported for NEE patients. The neural tube was not closed completely and the MHB was absent in morphants, which are comparable with neural tube defects in humans (Kao et al., 2014). In clinical cases, causes of neural tube defects in humans are associated with the imbalance of folate pools (vitamin B9). A key enzyme involved in folate metabolism is serine hydroxymethyltransferase, which uses tetrahydrofolate as a coenzyme and PLP as a cofactor. Deficiency of PLP may imbalance folate pools and result in brain malformation (Greenberg et al., 2011).

Increased locomotor activity, such as moving distance, turning angle, or acceleration, has been used to characterize seizure behavior in zebrafish (Pena et al., 2017; Zheng et al., 2018). However, in our zPnpo deficiency model, we did not observe such epilepsy phenotypes but rather observe increased spontaneous and erratic behavior at 24 hpf, with activity decreasing at 4 dpf, indicating neuron damage. One possible cause contributing to the differences in anatomical characteristics between B6 deficiency, reported for PNPO deficient patients and observed in zebrafish morphants, might be attributed to the maternal supply of active B6 continuing throughout the pregnancy for the fetus, which will not be possible for zebrafish embryos. However, we could not exclude the possibility of species differences, since developmental anomalies, including nervous system defects, anemic characteristics, omphalocele, exencephaly, cleft palate, micrognathia, digital defects, splenic hypoplasia, and impeded bone development, have also been reported for lab animals enduring chemically induced vitamin B6 deficiency (Davis et al., 1970; Kirksey et al., 1990; Masse et al., 1990).

We needed to confirm that the phenotypic results of the knockdown of zPnpo were not the result of off-target genetic changes. This was tested by injecting knockdown embryos with the mRNA for wild-type zPnpo. If off-target changes were responsible for the phenotypic observations, then at least some of the problems would still exist when normal levels of zPnpo were restored. We observed, however, that the mRNA for zPnpo restored the larvae to the wild-type phenotype (**Figure 5**). An even more encouraging result was that the mRNA for hPNPO also restored the wild-type zebrafish phenotype (**Figure 5**). The observation that injecting either human or zebrafish *pnpo* mRNA provides the rescuing effects as effective as the addition of PLP in alleviating morphant mortality and organ anomalies further supports knockdown specificity. This observation also permits future studies to test the different mutations in hPNPO on restoring the phenotype in the different organs of zebrafish. We are currently expanding our zebrafish research by knocking out the gene for zPnpo in addition to only using the knockdown. We note that backcrossing the knockout fish with wild type for a few generations has recently been reported to minimize off-target effects (Johnstone et al., 2019).

We next tested some of our ideas for new therapies. One idea was to use PM as a rescue agent. The conversion of apo-B6 enzymes to catalytically active holo-enzymes requires PLP, except for one family of apo-B6 enzymes. The largest family of B6 enzymes are transaminases that convert amino acids into the corresponding α-keto acids. The transaminases can also use PMP to form the holo-enzyme. We proposed that PM may also help restore at least some of the deleterious effects from decreased activity of zPnpo in our knockdown fish. Since the fish still contain a normal level of pyridoxal kinase, the PM would be converted to PMP and then activate apo-transaminases. As shown in **Figures 5** and **6**, this was observed when low levels of PM were used. The effect was less encouraging when large doses of PM (>1 mM) were used (data not shown). We believe that this is the result of the large doses of PM being converted to PMP by the kinase, which exceed the requirement for activation of all apo-transaminases. The excess PMP may act as a feedback inhibitor of pyridoxal kinase and block conversion of PL to PLP that is needed for activation of non-B6 transaminases (**Figure 1**). The fact that PLP and PM, in some instances, are able to rescue different anomalies implies the potential tissue specificity for B6 metabolism/requirement, and that combining these two B6 vitamers may reduce PLP dose requirements and mitigate potential toxicity. Future studies will focus on determining the optimal combination of PM and PLP as a rescue therapy for NEE.

A second possible therapy is to determine which apo-B6 enzymes are the most crucial to restoring the normal phenotype with the knockdown zPnpo fish. The many expressed apo-B6 enzymes will compete for available PLP. With the knockdown zebrafish, there will be some PLP available from PL kinase (reaction 1, **Figure 1**). Most likely, competition for the low level of PLP in these fish will not be the same for all apo-B6 enzymes. The apo-B6 enzyme that does not compete well will be left largely in the inactive apo-form. Adding a downstream product of this inactive apo-enzyme may help to restore the normal phenotype. We tested this idea by adding GABA, the product of γ-amino glutamate decarboxylase. GABA is a key neurotransmitter. As shown in **Figure 7**, addition of GABA did have a positive effect on larvae from zPnpo knockdown zebrafish, indicating a possibly lowered GABA level in morphants due to decreased expression of zPnpo. We are currently developing an assay to determine the competition of several key apo-B6 enzymes for PLP to determine which B6 apo-enzyme might be a problem in zebrafish with limited zPnpo activity. This will permit testing many downstream molecules as possible rescue agents.

## Data Availability

All datasets generated for this study are included in the manuscript/**Supplementary Files**.

## Ethics Statement

The animal study was reviewed and approved by Institutional Animal Care and Use Committee, National Cheng Kung University, Tainan, Taiwan (IACUC Approval No. 106086).

## Author Contributions

T-FF, MS, and VS conceptualized this study. P-YC and T-FF designed the experiments. P-YC and H-CT performed the experiments. P-YC, VS, MS, and T-FF analyzed the data and wrote the manuscript. MS and T-FF are responsible for funding acquisition.

## Funding

This work was supported by research grants (MOST106-2311-B-006-004-MY3) funded by the Ministry of Science and Technology, Taiwan, to T-FF and NIH/NIMHD grant MD009124 to MS.

## Conflict of Interest Statement

The authors declare that the research was conducted in the absence of any commercial or financial relationships that could be construed as a potential conflict of interest.

## Supplementary Material

Figure S1 **The reactivity and specificity of anti-zPnpo antibodies.** The cells extracts prepared from the *E.coli* with (+IPTG)/without (-IPTG) overexpressing the recombinant zPnpo used for antibodies generation was subjected to Western blotting with custom-made anti-zPnpo antibodies. *E. coli* strain HMS174(DE3) encompassing the plasmids Nus-zPnpo/pET43.1a was added with 0.6 mM IPTG in log phase and grown for 6 hours at 37°C. Approximate 5 ml bacterial culture was collected and sonicated. The supernatant of cell lysate containing 2.25 μg protein (per lane) was subjected to Western blot analysis.

Figure S2 **Whole-mount in-situ hybridization with *zpnpo* sense probe.** Embryos at the indicated stages were collected and subjected to WISH by probing with the DIG-labeled riboprobes complementary to the anti-sense strain of *zpnpo* coding sequence (sense probe) as described in Materials and Methods.

Figure S3–10 **The impeded heart development observed in zPnpo morphants.** Tg(*cmlc2*:eGFP) embryos with (3-6; MO)/without(7-10; WT) zPnpo MO injection were video-recorded at 3 dpf for heart pumping. Diminished red blood cells, decreased heart rate, distorted heart tube and incorrectly positioned cardiac chambers were observed in zPnpo morphants.

Figure S11 **The impeded trunk development in zPnpo morphants was rescued by co-injecting *pnpo* mRNA and supplementing with PLP.** Embryos injected with 5 ng zPnpo MO at 1 to 2-cell stages were co-injected with 800 pg *zpnpo* or h*PNPO* mRNA (columns 4 and 5), or grown in embryo water containing 1 mM PLP, 1 mM PN, 0.1 mM PM, or 0.1 mM PL (columns 6-9). The embryos displaying body curvature, representing mild defect (A), and lack of tail, representing severe defect (B), were recorded at 4 dpf. The Reported are the averages of data from at least three independent repeats with n = 60~80 embryos for each group and expressed as mean ± SEM.
