# Supplementary figures and images for: Pyridoxamine Supplementation Effectively Reverses the Abnormal Phenotypes of Zebrafish Larvae With PNPO Deficiency
Source: Front Pharmacol. 2019 Sep 20;10:1086. doi: 10.3389/fphar.2019.01086 (PMC6764245; doi:10.3389/fphar.2019.01086)

Suppl. 1

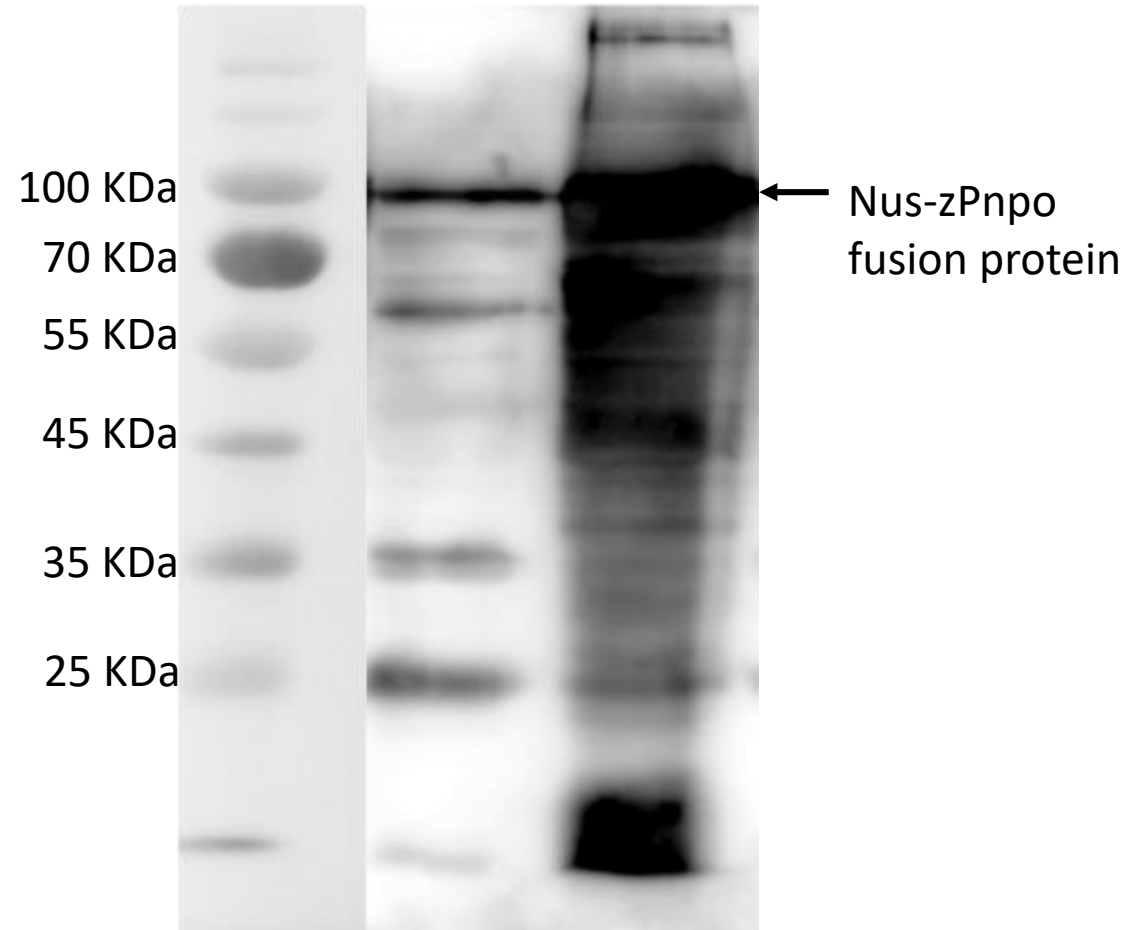

## Suppl. 2

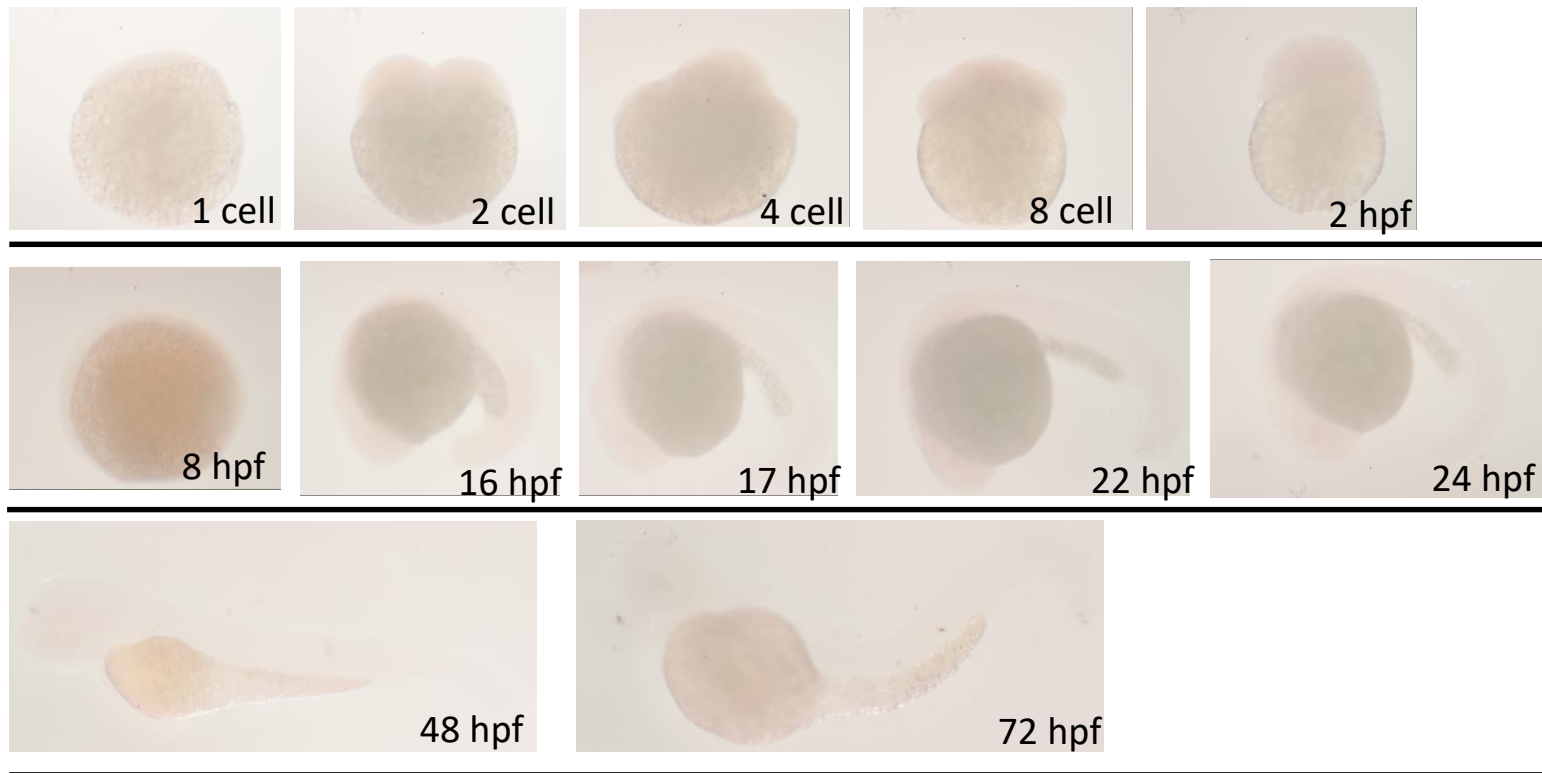

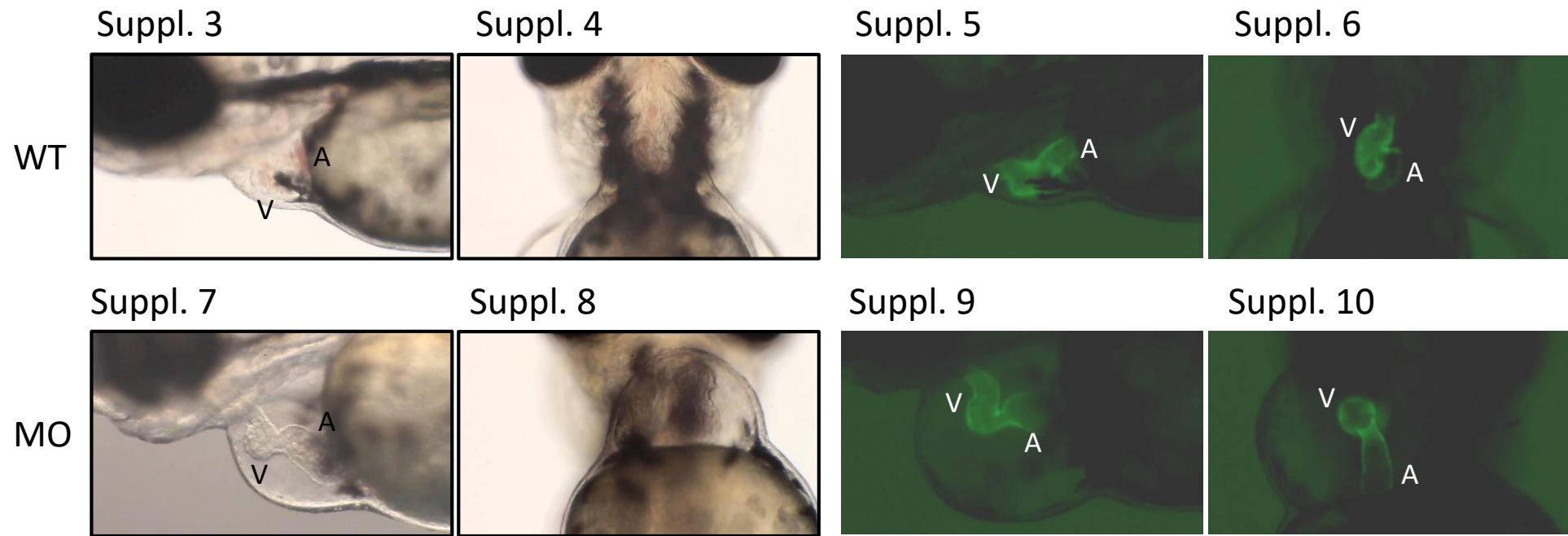

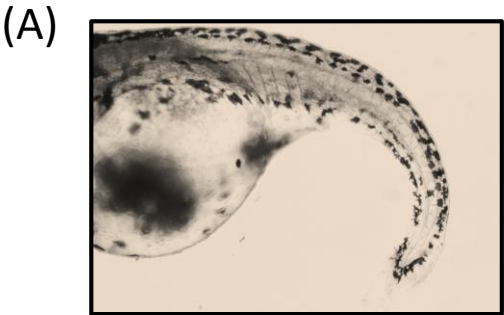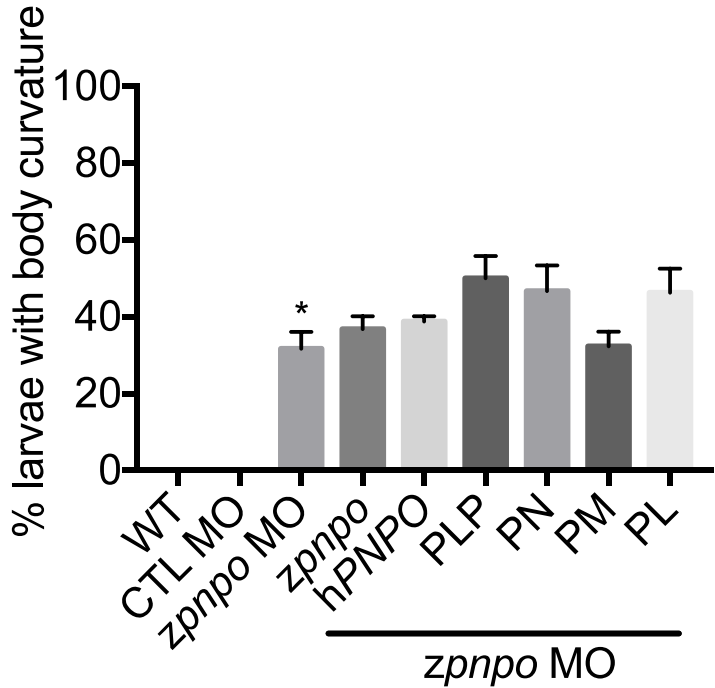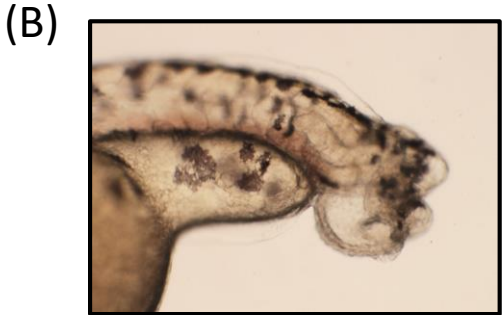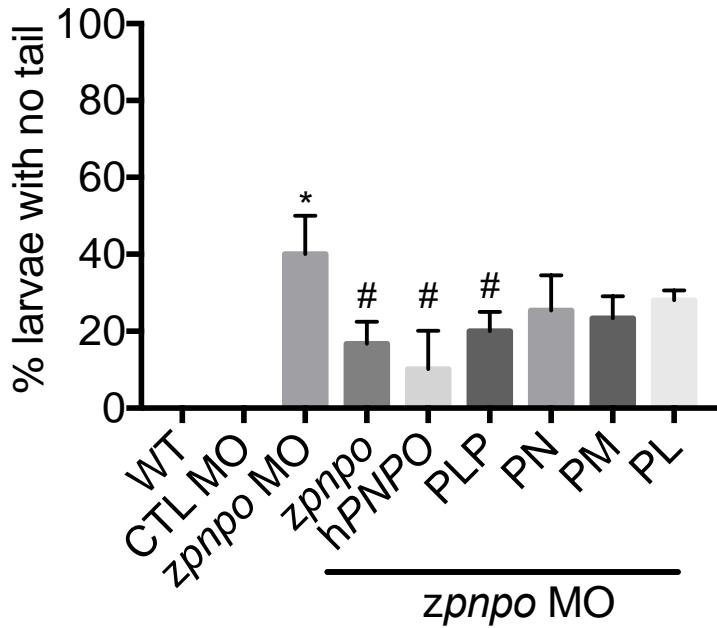

Supplement: Figure S1 — The reactivity and specificity of anti-zPnpo antibodies. The cells extracts prepared from the E.coli with (+IPTG)/without (-IPTG) overexpressing the recombinant zPnpo used for antibodies generation was subjected to Western blotting with custom-made anti-zPnpo antibodies. E. coli strain HMS174(DE3) encompassing the plasmids Nus-zPnpo/pET43.1a was added with 0.6 mM IPTG in log phase and grown for 6 hours at 37°C. Approximate 5 ml bacterial culture was collected and sonicated. The supernatant of cell lysate containing 2.25 μg protein (per lane) was subjected to Western blot analysis. [file DataSheet_1.pdf]
